# A qualitative study of factors influencing retention of doctors and nurses at rural healthcare facilities in Bangladesh

**DOI:** 10.1186/s12913-015-1012-z

**Published:** 2015-08-27

**Authors:** Emmanuel Kwame Darkwa, M. Sophia Newman, Mahmud Kawkab, Mahbub Elahi Chowdhury

**Affiliations:** Dodowa Health Research Center Accra Apetechi, Greater Accra Region, Ghana; Independent Scholar, Mohakhali, Bangladesh; James P. Grant School of Public Health, BRAC University, ICDDR, B Building, Sixth Floor, Mohakhali, Dhaka 1212 Bangladesh; Centre for Equity and Health Systems (CEHS), International Centre for Diarrhoeal Disease Research Bangladesh (icddr,b), Mohakhali, Dhaka 1212 Bangladesh

## Abstract

**Background:**

Bangladesh is a highly populous country with three-quarters rural population. Pressing national shortages in health professionals has resulted in high vacancy rates in rural areas. These are compounded by excessive absenteeism and low retention among nurses and doctors posted to rural locations. This study attempts to ascertain reasons for providers’ reluctance to work in rural and remote areas and to identify ways in which these barriers to appropriate staffing might be resolved.

**Methods:**

This is a qualitative study based on in-depth interviews with healthcare providers (*n* = 15) and facility managers (*n* = 4) posted in rural areas, and key informant interviews with health policymakers at the national level (n = 2). Interview guides were written in English and translated and administered in Bengali. The collected data were re-translated and analyzed in English. Braun and Clarke’s thematic analysis approach (data familiarization, coding, identifying and reviewing themes, and producing a final report) was used.

**Results:**

Participants reported poor living conditions in rural areas (e.g., poor housing facilities and unsafe drinking water); overwhelming workloads with poor safety and insufficient equipment; and a lack of opportunities for career development, and skill enhancement. They reported insufficient wages and inadequate opportunities for private practice in rural areas. Managers described their lack of sufficient authority to undertake disciplinary measures for absenteeism. They also pointed at the lack of fairness in promotion practices of the providers. Policymakers acknowledged unavailability or insufficient allowances for rural postings. There is also a lack of national policy on rural retention.

**Conclusions:**

The findings revealed a complex interplay of factors influencing doctors’ and nurses’ availability in rural and remote public health facilities from the perspective of different players in the healthcare delivery system of Bangladesh. In addition, the study generated several possibilities for improvement, including increased allowances and incentives for rural posting; a transparent and fair promotion system for serving in rural areas; enhanced authority of the local managers for reducing worker absenteeism; and improved national policies on rural retention.

## Background

Human resources for health are one of six building blocks of a health system [1]. Yet the world is facing a shortage of approximately 4.2 million healthcare workers (doctors, nurses, dentists and midwives) [2]. Globally, there is a close association between the concentrations of qualified health workers and key health outcomes, such as immunization and infant, child, and maternal survival [3]. Therefore, this gap is a fundamental constraint on achieving health-related millennium development goals [2].

People in rural areas are disproportionately impacted by human resource shortages. Ministries of Health and policymakers face significant challenges in meeting the health needs of rural populations [4] due to the shortage of healthcare providers, lack of human resources retention in rural areas, and inequitable geographic distribution of healthcare providers [5].

All countries, rich or poor, report a higher proportion of health personnel in urban and wealthier places [6–8]. Half of the global populations live in rural areas served by just 38 % of the nursing workforce and 24 % of the medical workforce [5]. Evidence alludes to healthcare professionals’ preference for the social, cultural and professional rewards available only in urban areas [9].

Bangladesh is the most populous of the least-developed countries, having a population of about 160 million with >70 % rural-dwellers [10]. Bangladesh suffers from both a shortage of healthcare providers as well as inequitable distribution of human resource for health (HRH) between rural and urban areas. Overall, there are approximately five physicians and two nurses per 10,000 persons, which is far short of WHO requirements [11]. A study observing the existing health workforce in Bangladesh in 2006 found that 35 % of doctors and 30 % of nurses were in four major cities (Dhaka, Chittagong, Rajshahi, and Khulna) where less than 20 % of the population lived [12]. Documents from the Ministry of Health and Family Welfare (MoHFW) put hard numbers on staffing shortfall. Currently in Bangladesh, there are 53,977 doctors, of whom 43,537 live in the country [13]. Only 35 % of this number work under the MoHFW [13]. The nursing population is also very low. Out of a total of 26,899 registered diploma nurses, an estimated 15,023 are currently serving the country. The medical doctor to population ratio in urban areas is 1 doctor per 1500 persons; in rural areas, the corresponding figure is 1 per 15,000 [14].

An imbalance in allocation of workforce favors urban areas and is exacerbated by internal migration and absenteeism in rural healthcare facilities [14]. The Joint Learning Initiative on Human Resources for Health [15] identified five challenges to human resources for health: overall shortages, imbalance in skill mix, inequitable distribution and migration of providers, a weak knowledge base, and a negative work environment. Bangladesh faces all five challenges.

In Bangladesh, *upazila* [sub-district] health centers (UHCs) were designed to provide a wide range of healthcare functions. They also play an important link in the referral system of healthcare delivery [11]. Yet vacancy rates are acute in rural UHCs. More than 25 % of physician positions and 22 % of nurse positions at UHCs are vacant. The vacancy rate is 52 % for specialist providers [16].

Bangladesh National Health Policy (2011) emphasizes the healthcare availability across all divisions, with particular stress on improving availability in remote and poorly served areas [17]. The country has been successful in placing health centers in all of its *upazila*s. However, this only increases the challenge of ensuring those centers are staffed.

In Bangladesh, the MoHFW has put in place strategies to enhance motivation and retention of health workers beginning in 2008. These strategies aim to develop packages of financial incentives for rural areas. Currently there is hill tract allowance to those health workers in hill districts of Chittagong, one of the seven administrative regions of Bangladesh. They also include a compensation package to enhance motivation and retention in public service [18]. Evidence suggests that appropriate financial incentives play a role in attracting and retaining health workers to rural areas [4, 5]. In Bangladesh, government has implemented a provision to provide a premium of 30 % of base salary for health workers in hard-to-reach areas, particularly in three districts in the Chittagong Hill Tracts. (Currently, standard salaries are 30,000-80,000 BDT per month for a non-specialist doctor [or US$384-1026, at an approximate exchange rate of 78 BDT: US$1] and 20,000 to 50,000 BDT per month [US$256-641] for a nurse. Specialist doctors earn widely variable salaries depending on their area of focus and seniority.) However, implementation has been poor, there is no provision for health workers in other rural districts, and the overall effect has been far less than the desired improvement. The 2013–2023 workforce strategy aimed to improve upon the 2008 one [19], but, at the time of data collection, had not yet been implemented fully.

That said, reasons for high absenteeism and poor retention remained largely unexplored. Thus there is a need to consider the reasons for providers’ disfavoring rural placements. This study attempts to identify qualitative factors that affect the willingness of doctors, nurses, and midwives to remain in rural and remote health facilities in a district called Sunamganj in Bangladesh, and to identify policy factors for provider availability at rural, remote and hard-to- reach health facilities. The findings and recommendations will be useful to governmental program planners, community health organizations, academic researchers, and providers.

## Methods

The study employed qualitative methods to explore and understand factors affecting the availability of doctors and nurses in a rural and hard-to-reach district of Bangladesh. The qualitative techniques for data collection were key informant interviews (KIIs) and in-depth interviews (IDIs), with an addition of document review of national policies relevant to rural retention. The qualitative study design was chosen because this study aimed to understand the emic perspectives of providers and policymakers on motivations and challenges of working in a rural setting, factors affecting retention, and policy factors for provider unavailability.

### Study site and time frame

Sunamganj, a rural and remote district in north-eastern Sylhet Division, is a low-lying area reliant on boat transportation during the rainy season due to its severe susceptibility to floods (locally termed a *haor*). Sunamganj was chosen out of 64 districts in Bangladesh because it is one of the low-performing districts in health indicators and human resource retention, and because it was also reachable at a time of severe political disruption, which affected the study as described below. The district has a vacancy rate of 47.7 % across all government-sanctioned health provider posts, including a 63 % vacancy for doctors [20]. This district has one district hospital and nine *upazila* health complexes. The main study sites were the district hospital and two *upazila* health complexes purposively selected based on their accessibility to the research team. Two different settings (district and *upazila*) were used to increase comprehensiveness of data. In addition, interviews were conducted at the Government of Bangladesh’s Directorate General of Health Services (DGHS) and Directorate General of Family Planning (DGFP) in Dhaka. The study data was collected in two weeks in late 2013.

### Protocol and tools development

The study protocol and tools were developed based on the objectives to understand factors affecting retention and policy factors for healthcare staff unavailability. For doctors and nurses, guidelines consisted of open-ended questions on economic, contextual (working and community) conditions and career-related issues affecting retention. We developed separate tools for providers and district managers and for policymakers.

### Study population and sampling

The study population was doctors and nurses in district and *upazila* government healthcare facilities in Sunamganj district; managers of healthcare facilities in the same district; and national policymakers. All of the 21 respondents were chosen purposively. The managers were chosen on the basis of their position as in-charge of the doctors and nurses in the district health facilities. At the district and sub-district level, we interviewed doctors and nurses with at least a year of working experience in the district, who were available and willing to participate. In order to ensure diverse perspectives on the issues, we purposively sought different providers, such as specialist doctors, general practice doctors, and nurses. Throughout, we focused on those who provided maternal, neonatal, and child healthcare (MNCH) services. Selection was also impacted by vacancies; the district hospital has 59 sanctioned post for doctors, but only 18 (30.5 %) were filled, and 190 sanctioned posts for nurses, of which only 56 (29.5 %) were filled. At the two sub-district level facilities visited, there were 25 sanctioned posts for doctors, of which 11 (44 %) were filled, and 21 sanctioned posts for nurses, of which 13 (61.9 %) were filled. We interviewed two specialists and two general practice doctors (4/18 = 22.2 %), and four nurses at the district levels (4/56 = 7.1 %). At the two sub-district facilities, we spoke to three doctors (3/11 = 27.3 %) and four nurses (4/13 = 30.7 %). The high vacancy rates cuts across all other sub-district level facilities, and therefore we believe our sample to be representative. The study also included four managers, two from the district level and two from the sub-district level, out of a total of nine working in the area (4/9 = 44.4 %). We also interviewed two national policymakers, who were purposively selected as a result of their work on the issues considered in this study. Student doctors under apprenticeship and policymakers not concerned with rural retention were excluded from the study.

### Ethical considerations

The study protocol was approved by the ethical review board of James P. Grant School of Public Health. The school provided a letter of introduction to all study sites. Each healthcare facility agreed to permit contact with providers and managers prior to researchers approaching them. Policymakers agreed individually to participate. Respondents were informed of study objectives, purpose, process, risk and benefits from participation, prospective uses of collected information, and anonymity and confidentiality of personal identifiers. This included exclusion of personal names, facility names, and location names where relevant (i.e., names of home villages mentioned by participants). Informed consent was taken from all respondents in writing prior to the start of the interview. They were informed that complete anonymity may not be possible due to the size of the study and disclosure of their occupation and location, and most participants expressed that, due to the study topic, anonymity was not a concern. They were assured that they could withdraw at any point in the interview.

### Translation of guidelines and pretesting

All guidelines were translated to the local language (Bangla) by a research assistant. The guidelines were back-translated to English to ensure consistency of meaning in all questions. Following this step, the research assistant received two days of training in interviewing and familiarization with the interview guide. The guidelines were pretested in Dhaka Medical College with one doctor, one nurse, and one supervisor. During pretesting, a tape recorder and handwritten notes were the main sources of data recording. The research assistant’s handwritten notes were later crosschecked against the recording. After pre-testing, some questions were rephrased and modified to ensure clarity.

### Practical challenges

The research was conducted at the time when the main opposition party in Bangladesh was conducting strikes (*hartals*) and road blockades countrywide. These occurred frequently during the last six weeks of the year 2013 for which transportation for data collection were inhibited. While the study topic was not viewed as a political issue under contention by the opposition party and the researchers themselves (particularly the foreigners) were considered neutral, interviews at both district and sub district-level facilities occurred amid general insecurity and fear. An interview scheduled with the Director of Nursing Services (DNS) could not been conducted due to repeated shutdowns of that office (and most other workplaces). Overall, the crisis caused a reduction in the number of days for data collection and an increase in the number of hours of work per day.

### Data collection: in-depth interviews

In-depth interviews were used to collect information from doctors and nurses at the health facility environment or in the residence of providers, based on their preference and time constraints. We conducted 15 interviews with providers (eight doctors and seven nurses). In-depth interviews explored doctors’ and nurses’ willingness to stay and work in rural healthcare facilities as per their job descriptions; the perspective of different kinds of providers (specialists, non-specialist doctors, and nurses); personal and job-related environmental factors that influenced rural retention; and possible solutions. Each interview lasted 40 to 60 min. Finally, some brief informal discussions held with doctors, nurses and some facility mangers were included in notes used to analyze the data. No interview was refused by any of the selected respondents. Data saturation was reached when doctors and nurses begun repeating the same responses from other interviews.

### Data collection: Key informant interviews

A separate key informant guidelines was developed for district managers and policymakers. Six key informant interviews were carried out with district and facility managers at Sunamganj (*n* = 4) and national policymakers in Dhaka (*n* = 2). Facility and district managers helped us understand factors affecting provider retention at district and *upazila* levels. Policymakers helped us understand policy factors for the availability of doctors and nurses at rural health facilities. Themes explored included current and future strategies for transfer, posting, recruitment, and supervision; and policy measures to ensure providers are available in facilities.

### Data collection: document review

We also reviewed national policy documents to complement the key informants’ interviews on policy factors. Although Bangladesh does not have a free-standing policy on rural retention, some policy documents have sections relevant to rural retention and HRH development. A tentative list of such policy documents was prepared and suggestions were taken from key informants to enrich the list. Academic databases, government websites, and grey literatures were searched. Content analysis of policy documents was completed and a summary of relevant information was included in results.

### Data management

Most participants consented to the use of audio recorder with the exception of four doctors and four nurses. Handwritten notes were used in these interviews instead. All information was transcribed every evening to minimize recall bias. During the transcription, the research assistant and lead author (EKD) often discussed important points and issues from the interviews. Interviews in Bangla were translated immediately into English. Notes from interviews were also expanded and entered immediately into Microsoft Word.

### Data analysis

Data analysis was done in different stages, adopting Braun and Clarke’s thematic analysis approach: data familiarization, coding, looking for themes, reviewing themes, and producing a final report [21]. A list of *a priori* codes was initially developed from the interview guidelines and was used to code the transcripts for analysis. Multiple readings of each individual transcript were done by the four members of the research team, and further analysis involved inductive codes generated from emerging key themes from data. Ultimately the data was arranged in clusters per *a priori* and inductive codes. The research team used intercoder reliability to evaluate the validity of the coding categories. Quotes extracted from the raw data provided evidence for each theme and categories extracted. Quotes were selected to illustrate the dominant opinion of participants (unless otherwise stated below). Data analysis was done manually in MS Word, without specialized software.

## Results

### Overview of respondents

A total of eight doctors and seven nurses from both the district and *upazila* facilities delivering MNCH services participated in the study. Most of the doctors were male (*n* = 7). The only female doctor participating was a specialist gynecologist at the district level (*n* = 1). The nurses were all female (*n* = 7). All positions for specialist doctors, resident medical officers (RMOs), and nursing supervisors were vacant at the two *upazila* facilities visited.

The study also included facility managers (*n* =2) and district managers (*n* =2) plus national policymakers (*n* = 2). All were male.

Only one doctor was not married among all the providers we interviewed. The rest were married with children. Of the fifteen providers (doctors and nurses combined), nine were originally from a rural area and the remainder were raised in or near a city.

### Providers’ overall attitude

Most respondents (*n* = 14/15) viewed the healthcare profession as honorable and believed a good healthcare worker would be well remembered for helping save lives. Some doctors (*n* = 5/8) and nurses (*n* = 6/7) mentioned that they joined this profession to be able to advance the health of families and communities. Many participants (*n* = 7/15) attached a very strong sense of duty to helping ones’ fellow humans, motivated by humanitarian and religious impulses: *“It is my duty to help my people. I managed my way to get posting to this place because it is my home district… I am proud to serve my people. Sometimes it is not all about the money but the joy of seeing my people happy. Even though we don’t have all equipment, we manage”* (Nurse, female).

At all levels of healthcare, there were some providers who reported strong dedication to their rural postings. Most (*n* = 9/15) come from the area or made a decision to settle there. Professional expectations and the joy of serving one’s home district were more likely to have been a crucial reason for choosing and maintaining a rural posting. *“I joined the profession to eventually come to help my home district one day. The district is very poor and my colleagues do not want to come or stay here…everybody is fighting to go to the big cities or to their home districts as well…you see we are fond of our families very much.”* (Specialist doctor, female)

However, at both district and *upazila* levels, most respondents (*n* = 13/15) described having been assigned, rather than having chosen, rural posts. While several of the nine providers with rural backgrounds expressed interest in serving rural areas (including specialists, non-specialists, and nurses), other providers expressed anxiety about the unfamiliarity of rural life, concern for their children’s education, and concern for their careers and professional development.

### Career and professional development

Career development was identified by the overwhelming majority of doctors, nurses, facility managers and district managers as well as the policy personnel from DGHS and DGFP as the most critical hindrance for doctors’ and nurses’ willingness to work in rural areas. Opportunities for career progression and further training are structured to favor those working in big cities. All doctors and nurses interviewed (*n* = 15/15) describe cities like Dhaka, Chittagong, Rajshahi and Khulna as the best places to work and access postgraduate or specialist training, study leave or international opportunities, as well as mentoring or coaching by specialists or senior doctors. “*I want to do BSC [Bachelors of Science] in nursing for my promotion, but it is not available in this district. I wonder how long I can stay here. You see this is one reason people are running from this area.*” (Nurse, female).

Even at the district level, which provides a few more urban amenities, the providers (*n* = 7/15) alluded to the difficulty in pursuing professional development. *“I was admitted in a post-graduation course in Dhaka (the capital city). Being transferred here, I was not able to continue my study.”* (Non-specialist doctor, male)

Most providers repeatedly linked sluggish career progression to post vacancies. With many positions vacant, they were forced to take on a heavy workload. The sheer volume of work made it nearly impossible to consider any new opportunities, such as education, beyond the current demands. “*Sometimes when you come here it becomes difficult to progress; you go back to the medical colleges and all your colleagues are far, far ahead of you"* (Non-specialist doctor, male).

Doctors and nurses had inconsistent answers about current policies on professional development with regards to rural postings and reported that they thought incentive structures were not in current use or they were unsure about such structures. *“There are no laid-down opportunities to work in rural areas… [For example], ‘Once you accept postings to a rural and remote area say for three years, these are the plans for you; you can be sponsored to go for a post-graduate program.’ But these things are not there…. You have to struggle to make it and this is not the right place. You have to be in the cities”* (Non-specialist doctor, male).

### Working conditions

Working conditions were described as difficult and the MNCH workload as particularly heavy. This was attributed to the shortage and unavailability of trained staff. *“We work more than expected because there are no doctors to run shifts. Here I am the only Gynecology consultant so you can imagine the work load. I have a lot of pressur*e.*”* (Specialist doctor, female)

Most doctors and nurses (*n* = 14/15) reported having good relationships with community members, but one nurse reported having a confrontation with the families of a patient who claimed she had killed their daughter. She was unhappy about the people’s behavior, and was considering transferring from the district. *“They brought the woman here almost unconscious after they tried to deliver her at home but they failed. She had lost so much blood and died a few moments after arrival. I’m not happy here.”* (Nurse, female)

Overall, nurses (*n* = 3/7) reported serious concerns about occupational hazards at *upazila* health complexes (but not at the district level). “*Here we work under pressure. We have to manage public affairs. But in cities there are so many ward boys. At night there is no security here… Sometimes we feel very insecure at night duty… Doctors are not available at night. If patient comes at night with any serious condition, relatives of patient misbehave with us. Sometimes they try to beat us. That time we have nothing to do. Ward boys are not available here. But in cities there are many nurses and ward boys at duty in the night. They can control any type of problem.” (*Nurse, female)

### Hospital infrastructure and equipment

In addition to the points above, doctors and nurses raised the issue of inadequate equipment and facilities. Some facilities require renovation. Staff commented on their viewpoint that hospitals in larger cities are better equipped. “*In the urban areas doctors get more modern equipment and facilities to work with. They can do any type of operation and treatment. Here we don’t have, though we have the skills, we cannot put our skills [to use] here…We faced so many hazards to do our duty.”* (Specialist doctor, female)

### Financial incentives

Most doctors and nurses (*n* = 12/15) argued that rural service deserved higher financial incentives, due to the higher workload, poor working conditions, and lost opportunities for supplementary income from private practice. This view was strongly shared by specialist doctors who emphasized how tough it was to work without adding a private practice. Many felt that the salary package had to improve to attract specialist doctors into rural areas. "*My colleague in Dhaka gets the same salary as I draw here. But they go to work at 8 am and then … at around 2 pm they close, so they can go to a private hospital and do private practice. But here the whole day I am in the hospital. If they (MoHFW) want to be fair and keep us here, then we need some extra top-ups to our salaries to balance the playing field with our colleagues in the cities.***”** (Specialist doctor, female).

Facility managers and district managers (*n* = 4/4) agreed that lack of opportunity for private practice affected retention and availability of doctors, especially the specialist. Some were unconvinced that salary alone would provide adequate incentive, but the dominant opinion was that extra financial incentives should be provided to flatten the already uneven playing field between urban and rural settings. “*There is no motivation to stay in rural areas, not at all. Our colleagues in Chittagong get a hill tract allowance… We are here in a* hoar *area which is difficult to live in, but we get nothing… This is unfair.****”*** (Specialist doctor, male).

### Accommodation and living conditions

At all the levels of health care visited, there were some sorts of government housing facilities available for doctors and nurses. All participants reported they had access to electricity but with constant power outages. But there was disappointment among some providers, especially the senior doctors and those with family, about the undesirable size, number, state of repair, furnishings, and social status implied by available housing. “*There are government quarters for me. But the condition is not good enough for me to live. I got some house rented with my salary.”* (Specialist doctor, male). The specialists (*n* = 2) and two of the non-specialist doctors have rented outside the facility whilst the remaining doctors (*n* = 4/8) say they are managing in the existing accommodation.

Finally, another challenging domestic factor has to do with lack of learning environment for their children which is one reason for not wanting to stay in a rural district with their families for longer periods. Seven providers (out of fifteen) shared this concern. “*I want the best education for my son but that quality is lacking here. So I have to send my family back to the city. It is not easy to stay here without my family…I try to visit them fortnightly which is quite challenging because of the distance involved.”* (Non-specialist doctor, male)

There were also concerns about the source of drinking water. Water was mainly from tube wells. Respondents from one *upazila* (*n* = 3/15) said that their water was arsenic-contaminated and unhealthy for drinking. *“There are so many problems and challenges working here in this place. There are no good schools here for our children, no good place to send the children for recreation, the water is arsenic [−contaminated], and the facilities here are mostly outmoded and need replacement. You see I have been here for just a year but don’t think I will be here for long. I had to send my wife and kid back to the city. That is the truth.”* (Non-specialist doctor, male).

### Managers’ perspectives

Managers (*n* = 4) confirmed the workload on staff and inadequate housing. *“According to government rules they have to do eight hours duty. But every so often they perform more than eight hours. For example in the Mother and Child Welfare Centre [MCWC] there is only one doctor. It’s a clinic. He has to do 24 h duty… If delivery patient come at 3 AM at night, he has to serve her. It is difficult for them.”* (District manager) *“Accommodation is not available for all doctors and their families. Most of the doctors live in rented house which they have to look for.*” (Facility manager, male)

They also noted transportation difficulties. “*Here is a* hoar *area and is prone to flood when it rains because it is a low lying area. It becomes difficult to move. The only means then is to travel by boat, which is not that safe.”* (District manager, male)

They described various other factors that affected retention. *Upazila* facility managers (*n* = 2) described how under-qualified staffs were used to fill gaps and help reduce MNCH workload. “*Sometimes, some experienced health assistants do the work of nurses because of the severe shortages and excessive workload on the few nurses we have.”* (Facility manager, male).

Another factor was the limited power of managers to take disciplinary measures against the staff who remain absent. The district health manager (a civil surgeon, who is an administrative head of district and sub-district facilities), other administrative authorities, and *upazila* health and family planning officers often lack adequate power to hire, fire, or transfer a doctor or nurse for absenteeism. The most they can do is to send a recommendation to the central directorate of health. “*The doctors who remain absent usually have good relations with the higher authorities and politicians. So sometimes when we write to the higher authorities actions are not taken always. It goes on for years often to take action and replace them.”* (Facility manager, male)

The lack of adequate power to take action against absent doctors exacerbates the unavailability of doctors. *“We don’t have enough power to punish at this level, everything is centralized in Dhaka. We can only send report of doctors and nurses and sometimes it takes forever for action to be taken. Some doctors have connections at the ministry so they do what they like while their post remains unattended.”* (Facility manager, male)

Another factor has to do with lack of power to motivate by rewarding performance at the facility level, since all this is done via recommendations to the central directorate level in Dhaka. *“Directly there is no reward system for better performance. But in the annual confidential report, he or she gains a good remark. For this, sometimes they get promotion. For nurses there is the president prize for better performance. But all these rewards come from the central level.”* (District manager, male)

The issue of retention also affects managers, who do not want to stay at rural locations. “*Some facility managers posted here do not want to stay here. How can they ensure the doctors and nurses stay? Look at the list. I am the longest-serving person since this hospital was established. I have been here for three years.*” (District manager, male)

### Policymakers’ perspectives

#### Overall shortage

Currently, Bangladesh is not producing adequate number of nurses and doctors nationally. The country’s capacity for educating providers is low, causing overall shortage. *“Bangladesh is currently not producing enough number of doctors and nurses…. annually approximately six thousand doctors and 3000–4000 nurses are being produced in Bangladesh at present. But this is not sufficient. There should be 3 nurses for one doctor.”* (Policymaker, male).

### Lack of policies

Respondents (*n* = 6) confirmed that there is no specific rural retention policy for doctors and nurses in Bangladesh. However, policymakers referred to government publications relevant to rural retention: policies on financial and non-financial incentives, career and professional development, effective supervision and monitoring, provision of necessary facilities and equipment at all facilities to create enabling environment for work.

### Financial and non-financial Incentives

In Bangladesh there is a provision to provide financial incentives to health workers, especially those working in rural areas. There is currently a policy to provide about 3000 taka (approximately US$36) to health workers in three hill districts in Chittagong division. In conversation, policymakers suggested that this allowance is inadequate and does not encourage retention and moreover the policy is not implemented in other rural and remote districts.

### Career and professional development

Policymakers confirmed that post-graduate programs are important to professional development for nurses and doctors, yet such opportunities are meager at the district and *upazila* level. *“Yes, there is career development pathway for doctors and nurses. If doctors complete their post-graduation degree, then they will become a consultant and get promotion rapidly. For nurses who have diploma degree [and] complete BSC course, then they will be promoted to nursing supervisor. But there is no opportunity to have these courses by staying in rural areas.”* (Policymaker, male).

However, administrative positions are accessible without post-graduate qualifications. *“There is no barrier to get an administrative promotion. But if one wants to do post-graduation then it is not possible from rural areas. Without this, there is no problem for career development*.” (Policymaker, male).

### Lack of fairness in promotions

As noted by doctors and nurses, promotions to administrative positions are not fairly distributed. The policymakers from DGHS and DGFP explained that there is a policy for a compulsory two-year rural service for all graduating doctors joining the public health service. However, unfair recommendation are given to doctors and nurses by politicians and senior government officials. Some do not serve the mandatory two years of rural posting, but rather receive transfers to urban areas and quick promotions. “*Performance is not sometimes the basis of transfer and promotion, rather unfair recommendation is the incentive here.*” (Policymaker, male).

### Document review

Policymakers referred to current policies on rural retention. There is no policy for rural retention of doctors and nurses in Bangladesh, but some national documents have some sections relevance to rural retention and HRH development. These include the Bangladesh Health Workforce Strategy 2008, Health Workforce Strategy 2013–2023 (draft), Health Policy 2011, and the MoHFW Human Resource Development (HRD) Data Sheet 2011 [17, 18, 22]. To complement these, we non-exhaustively interviewed policymakers from the DGHS. This revealed the following factors affecting provider availability.

The Bangladesh Health Workforce Strategy of 2008 and 2013 develop clearly specified career development plans for health workers. But these policies make no provisions on how the doctors and nurses working in rural areas can access such opportunities. Health Policy 2011 has sections that aim to ensure availability of adequate number of healthcare providers throughout the country; There was also the maternal health voucher program (Demand Side Financing, or DSF) developed by the MOHFW to increase the utilization of quality maternal health care services particularly by poor women. This program implemented through a cash subsidy to pregnant women to cover transport for antenatal care, institutional delivery and postnatal care. The program also provided incentives to providers in selected *upazila*s in Bangladesh. However, the incentives provided through the voucher system did not motivate public providers to offer higher level of services and it only covered 5 sub-districts of the country [23].

The government has published plans to increase the number of health providers by end of 2016. Targets are ambitious and promising, but achievement will require significant effort and commitment from the government, private sector, NGOs, donor partners and others.

## Discussion

This study confirmed previous findings on the importance of financial incentives, local recruitment, and career development opportunities as important factors for retention of health care providers in the rural settings. In addition, this study adds insights into absenteeism, occupational risks, and loopholes in policy implementation.

### Monetary incentives

Monetary incentives are an important motivator for worker retention, especially in countries where government salaries are insufficient to meet basic needs of health workers and their families [24–27]. The blend of fiscally sustainable financial incentives, such as hardship allowances, free transportation, paid vacations, etc., are seen by health workers as sufficient to offset the opportunity costs associated with working in rural areas [5]. Improved salaries and benefits incentivize workers to remain in rural and hard-to-reach areas, and in some cases to give up competing private practices [28–30]. However, a study of six developing countries [31] found only 24 % of respondents endorsed financial remuneration as a condition on accepting rural postings. Our study confirmed that other factors are as important, as detailed below. In this study, doctors and nurses also argued that rural service deserves higher financial incentives because of lost opportunities to supplement income with private practice. Specialist doctors emphasized how tough it is to work without the opportunity to supplement income in the district. There were number of references of health workers receiving some allowances for serving in the hilly rural districts of Chittagong, one of the seven major divisions of Bangladesh. This is a policy in Bangladesh to reward providers for serving in hill districts with about 33 % enhancement of their basic salary. However, some policymakers commented that the amount is too small to help with provider retention. Increasing the incentive and applying this model to other districts may help with retention. Similar efforts in Vietnam and Thailand have been mostly successful [29, 30]. Several studies point to salaries and allowances as two of the key factors that influence health workers’ decisions to stay in or leave a rural workplace but our findings show that there exist some forms of allowances in some rural districts of Bangladesh, but it’s not enough. It’s moreover not implemented in some equally rural areas, which negates the WHO guidelines for health worker motivation in rural areas.

### Recruitment

This study found that those providers originating from the district of Sunamganj were willing to work in the district to help their families and neighbors. There is a convincing body of evidence from both poor and rich countries that a rural background increases the chance of graduates returning to practice in rural communities [5]. Some other studies have shown they continue to practice in those areas for at least 10 years [32–35]. A Cochrane systematic review states, “It appears to be the single factor most strongly associated with rural practice” [35]. As it has been shown that health professionals with a rural background are more likely to accept rural postings [4], some longitudinal studies following physicians in the USA have found that students with a rural background continue to practice in rural areas for an average of 11–16 years after graduation [5]. Similarly, Pathfinder Bangladesh in their 2004 study of staff of six clinics found that it was important to recruit providers close to their area of posting. In this way, family and relatives would provide support for rural providers and help improve retention [36].

### Career development

In Bangladesh, there are two career pathways for a person trained as a government doctor: pursuing a post-graduate degree to become a specialist, or promotion to an administrative position without a post-graduate degree. Most doctors and nurses interviewed preferred a post-graduate qualification, because they perceive that administrative promotion lacks fairness and transparency. No opportunities for post-graduate training exist in Sunamganj and other rural areas. Policymakers could address the attrition of doctors and nurses from rural areas by offering assistance with post-graduate admission or tuition, in exchange for a contracted promise to return to a rural area as a specialist for a set number of years upon degree completion. One of the recommendations of the WHO guidelines for rural retention is for the development of a career development program. In addition, senior posts in rural areas can help health workers move up the career ladder through experience, education and training, without necessarily leaving rural areas [5]. Previous research in Kenya found that the chance of getting health sponsorship and scholarship made work in the public sector more attractive, even though working conditions were better in the private sector [37]. Implementation of such a policy must be strictly adhered to promote trust in the system among doctor and nurses. Additionally, creating transparent practices that eliminate corruption in administrative promotions would encourage providers to entrust their career development to this path. Further research and testing is necessary for this suggestion.

### Absenteeism

Workers described extremely high workloads as a result of absenteeism on the top of vacant posts. At the district hospital, for example, there was only one gynecologist. At one *upazila* health complex there was only one medical officer providing MNCH services. The excessive workload burns out providers and entices them to take days off without permission (or to leave the area altogether). This in turn dumps their workload on the providers who do attend work, provoking them to burn out; the absentee problem is therefore a vicious cycle. In this study, respondents recommended breaking the vicious cycle via a system in which managers can hire, fire, and punish for absenteeism, including removing opportunities for corruption by moving control to the district level.

### Occupational risks

Moreover, serious concerns about the occupational risks of their work were reported by the two sub district level providers in this current study. This was mentioned particularly by nurses at the *upazila* level whilst nurses at the district level did not report this problem. This was a result of incidents in which patients and their families try to abuse them at night when other staff persons are not present. Violence against nurses seems to be a growing phenomenon. In review of global literatures by the International Council of Nurses on nurses retention and recruitment, indicated a link between work aggression, burnout and staff turnover [38]. The health care sector leads work sectors for nonfatal assaults, accounting for nearly half of all nonfatal injuries from violence against workers as of 2001 [39]. A study of 8,780 nurses at 210 hospitals in two provinces of Canada found that 46 % reported experiencing ≥1 types of violence routinely, including being spit on, bitten, hit, pushed, or subjected to verbal abuse [40]. Reducing danger at work can reduce provider migration [38]. This can be done by hiring local, non-medical people to act as security guards or even orderlies, particularly for night shifts with few or no doctors consistently present. Workplace violence prevention can be complex and costly. In resource-constrained settings, some steps towards safety are possible, including requiring workers to work in teams, community watch and alert systems, improvements to the layout of health facilities, and so on.

### Policy factors

Bangladesh has no specific policy on rural retention for career and professional development, financial and nonfinancial incentives and provision of adequate equipment for rural facilities. Although sections of some more general policies are related to rural retention, these policies have not been fully implemented. The WHO recommends that any retention strategy should be linked to the broader national and local health system structures and policies, to take advantage of synergies and increase efficiencies [5]. The situation in Bangladesh demonstrates the validity of those viewpoints, as the country has some general policies related to rural retention but incomplete implementation. Integrating retention strategies into a costed, validated national health workforce plan could help bridge the gap to implementation and effective impact. For example, it is compulsory for every newly recruited medical doctor to serve at least two years at the union health sub-center level, which is also a pre-requisite for a better career, particularly via eligibility for postgraduate training in medicine [41]. In addition, there is a need for new or improved policies on protecting staff from violence, adding strategies for reward and punishment, and introducing performance-based incentives. Although not specifically raised by any respondent, a national maternal health voucher program has some components meant to incentives providers in some selected upazila health complexes. However, the incentives provided through the program did not motivate doctors enough to offer higher services [23]. Programs like these could also be improved. Every government influences the health labour market through regulations and policies as an entirely free labour market will never lead to a well distributed health workforce [5]. In China, Cuba and Thailand for instance, long standing commitment towards education, trainings and specific support to rural health workers have led to improve access to community health workers [5, 29, 42].

### Living conditions

Most staff reported living around the health facilities or within walking distance. Most also had electricity, although housing conditions were overall poor. This was true at the district and *upazila* levels. A study in Bangladesh discovered that remoteness and difficult access to health centers were major reasons for health worker absenteeism, and health personnel in areas with roads and electricity were far less likely to be absent [1]. Urban centres offer more opportunities for professional development, better employment prospects for family members, access to private practice (an important feature in countries where public salaries are low), social amenities, and access to better education for their children [11]. In addition, those in rural and remote areas face poor supervision and support [26–28], poor working conditions and lack of career development [27, 28, 43] and inadequate drug and equipment supply and communication [25] and excessive workload [44]. Our study confirmed the general idea that inadequacies in living conditions increase job dissatisfaction and the potential for migration. Improvement of rural health infrastructure and living conditions of the health care providers can have positive impact on retention in rural health facilities. Evidence suggests that having the basic infrastructure available and better living conditions can have significant impact on attracting health care providers working in the rural areas [4].

### Limitations

A language barrier contributed to loss of some information during data collection. Trust was reduced by the lead authors’ foreign appearance (he is African), as is unfortunately rather typical in Bangladesh. A perception of difference between Bangladesh and his country (Ghana) limited the amount of data that was collected from some respondents, as they wanted to avoid telling the truth of their predicaments to a foreigner. However, it is worth noting that political conditions in the country likely did *not* impact the information disclosed, as the topic of healthcare and rural staff retention have not association with widespread political conflicts.

The study was limited in terms of ascertaining the degree to which other districts may vary; however, respondents repeatedly voiced that the issues were national, and that variances between different rural districts were merely a matter of degree.

Another limitation to this study was that the exceptional rate of vacancy at the *upazila* levels. The posts of nursing supervisors and specialist doctors were all vacant. While this communicates a strong message about the issue of retention, the perspectives remain opaque on what personnel would occupy these posts, were they to be occupied. Additionally, focus group discussions of those personnel at most facilities visited could not be organized due to critical shortages in staffing. True data saturation could hinge upon reaching out to the people who have refused or avoided being present in rural postings, in addition to the relative minority who are present. This effort was beyond the scope of this study.

## Conclusions

The findings revealed a complex interplay of factors influencing doctors’ and nurses’ availability in rural and remote public health facilities from the perspective of different players in the health delivery system of Bangladesh. The motivational factors for attraction and retention at both district and sub district levels of health care were similar, although there were slight differences by provider type. Generally, we found that addressing rural health workforce shortages will require the development of a comprehensive, evidence-based strategy that accommodates financial incentives, career building opportunities, more delegated power to managers, and championing of policies by policymakers.

The Bangladeshi health system has particular challenges in maintaining a balanced distribution of doctors and nurses and minimizing overall shortage of doctors and nurses. The challenges in maintaining an adequate health workforce at both urban and rural settings requires a sustained effort in workforce planning, development and financing. The government of Bangladesh is pursing measures to scale up the production of more doctors and nurses in the country, including devising strategies to encourage and motivate health workers to stay and work in rural, hard-to-reach and disadvantaged areas as recommended by the WHO guidelines. This study suggests that improved career development opportunities, incentives, and provision of better working conditions will enhance retention of providers in rural and remote areas. Besides, policy concerns such as career progression plans, fair promotional policies, transparent system for posting and transfer, empowerment of local management for good governance will be critical to success.

## Abbreviations

DGFP: Directorate General of Family Planning
DGHS: Directorate General of Health Services
DNS: Directorate of Nursing Services
IDI: In-depth interview
KII: Key informant interview
MoHFW: Ministry of Health and Family Welfare
